# SpecTest: Specification-Based Compiler Testing

**DOI:** 10.1007/978-3-030-71500-7_14

**Published:** 2021-02-24

**Authors:** Richard Schumi, Jun Sun

**Affiliations:** 8 Universidad Autónoma de Madrid, Madrid, Spain; 9 University of Twente, Enschede, The Netherlands; Singapore Management University, Singapore, Singapore

**Keywords:** Mutation testing, Compiler testing, K framework, Formal semantics, Rare language features

## Abstract

Compilers are error-prone due to their high complexity. They are relevant for not only general purpose programming languages, but also for many domain specific languages. Bugs in compilers can potentially render all programs at risk. It is thus crucial that compilers are systematically tested, if not verified. Recently, a number of efforts have been made to formalise and standardise programming language semantics, which can be applied to verify the correctness of the respective compilers. In this work, we present a novel specification-based testing method named SpecTest to better utilise these semantics for testing. By applying an executable semantics as test oracle, SpecTest can discover deep semantic errors in compilers. Compared to existing approaches, SpecTest is built upon a novel test coverage criterion called semantic coverage which brings together mutation testing and fuzzing to specifically target less tested language features. We apply SpecTest to systematically test two compilers, i.e., the Java compiler and the Solidity compiler. SpecTest improves the semantic coverage of both compilers considerably and reveals multiple previously unknown bugs.

## 1 Introduction

Compilers must be thoroughly tested (if not verified) for multiple reasons. First, compilers are essential for the software ecosystem. Their correctness is a prerequisite for program correction. That is, a compiler bug might propagate to all produced programs. Second, compilers are error-prone due to their high complexity. Their main functionality is to convert source code to executable machine code. They often provide additional features, like code optimisation or debug utilities. A variety of compilers has been written for countless languages. Modern compilers like GCC, javac, and LLVM are overwhelmingly complicated (e.g., GCC has more than 7M lines of code and OpenJDK has more than 11M [20]). Although some of them have been used for decades, they may still be buggy [54,55].

Recently, there have been numerous efforts on formalising and standardising programming language semantics, such as K-Java [24], C semantics [29], KJS [47], or KSolidity [34,44], which readily serve as a specification of the respective compilers. Usually, these executable semantics are accompanied by manually crafted unit tests. Such tests are however designed to test the semantics rather than the compliance of the compiler to the language semantics. In this work, we aim to better utilise these semantics by automatically generating test programs with a novel coverage criterion that facilitates systematic compiler testing.

Multiple approaches have been recently proposed to test compilers. Most of them successfully found compiler bugs. For instance, the EMI project discovered more than 1600 bugs in GCC and LLVM [53]. Another study has revealed bugs in the Java compiler by comparing different javac and JVM versions [27]. For the relatively new Solidity (smart contract) language, many crashes were found through fuzzing [28]. Moreover, bugs in compilers may be exploited by attackers. For example, prior to version 0.5.0, the Solidity compiler had an uninitialised storage pointer vulnerability that affected many smart contracts on Ethereum. A honey pot named *OpenAddressLottery* was designed to exploit this vulnerably and steal ether (i.e., digital money in Ethereum). There are hundreds or even thousands of programming languages according to different sources [30] and many new ones emerge every year. For example, various new general purpose or domain-specific languages have been developed recently, such as Rust, Kotlin, Solidity, and Move.

Compiler testing is an ongoing research field. Next, we briefly review existing approaches according to how they address the following two problems.

1. The test generation problem: how are test cases (i.e., programs with specific inputs) selected and generated?
2. The oracle problem: how are testing results deemed successful or failure?

Existing compiler testing approaches solve the test generation problem mainly through two ways, by generating programs according to a grammar that specifies the syntax of a language [49,31,23], or by mutating existing seed programs [40,55,41]. For the former, due to a huge search space, additional selection criteria must be applied to selectively generate test cases for compilers, such as standard code coverage criteria like statement coverage. For the latter, existing mutation strategies are often limited by the 'weak' oracles (as we will discuss shortly) employed by the approach, e.g., mutating to introduce 'dead' code. Generally, approaches which generate complicated syntax focus more on parsing errors instead of errors in the semantics. For the oracle problem, existing proposals mainly have three oracles. The first oracle is one that only flags a test failure if the program is incompilable or leads to crashes [28]. The second oracle flags a test failure if certain algebraic properties are violated. For instance, the algebraic property adopted in the EMI approach [55] is that mutating unreachable code does not change the execution result. We remark that these two oracles are 'weak' as they are unable to detect simple semantic errors such as $3+4=8$. The third, stronger oracle is one that checks whether the output of a test program is consistent with a reference, which could be a second compiler (i.e., differential testing [45]), or an abstract specification like a state machine [35,36]. This oracle requires a reference, which is not always feasible. Furthermore, it is limited to bugs which result in inconsistencies between the compiled program and the reference. Last but not least, existing approaches do not provide a good adequacy measurement on the progress of compiler testing. Often measurements, like code coverage, are used as an indicator, but they have the limitation that they need access to the compiler code, and achieving full code coverage is challenging.

In this work, we present a novel specification-based testing method called SpecTest for compiler testing. SpecTest differs from existing approaches in the following aspects. First, SpecTest is built upon a strong oracle, i.e., an executable language specification that can predict the expected output of test programs. This strong oracle enables us to detect semantic errors, i.e., bugs that are related to the semantics. Such bugs may also originate from the runtime environment. Hence, SpecTest is not just limited to classical compiler bugs. Second, SpecTest offers a testing adequacy measurement in term of semantic coverage and has a built-in mutation-based test case generation method which aims to achieve high semantic coverage. The semantic coverage measures the number of language semantic rules that are covered by existing test cases. The test case generation method mutates the seed programs accordingly to maximise the coverage of the language semantics, e.g., by introducing less-tested language features into these programs. Compared to measuring the code coverage of a compiler, our semantic coverage has the added value that it does not need access to the compiler code, and it specifically targets semantic bugs.

Given a language semantics (in the form of a set of small-step operational semantic rules), SpecTest executes fully automatically. We have implemented SpecTest for two compilers, i.e., the Java compiler and the Solidity compiler and tested the language features that are supported by our applied semantics [24,44]. The results of the evaluation were promising. SpecTest successfully increases the semantic coverage for both compilers, and identified many bugs and issues that helped the compiler and specification developers.

To sum up, we make the following technical contributions.

- We propose a semantic coverage criterion for measuring the adequacy of compiler testing.
- We introduce a novel compiler testing method that uses an executable language specification as an oracle.
- We demonstrate the applicability and generality of SpecTest by applying it to two compilers.

The paper is structured as follows. Sect. 2 explains our method and discusses the required components in detail. In Sect. 3, we present our evaluation with two compilers. Next, we review related work in Sect. 4 and conclude in Sect. 5.

## 2 Method

In this section, we outline how SpecTest works. In particular, we present its high-level design, highlight relevant details of its components, and explain the workflow step by step using an example.

### 2.1 Overall Design

The overall workflow of SpecTest is depicted in Fig. 1. In the following, we introduce the tasks briefly before diving into the details of the main components.

(1) A set of user-provided seed programs are given as input to ***a program fuzzer*** one by one, which generates a set of test inputs for each program with the intention to cover as many program paths as possible. A program and the associated test inputs form a test case that is the basis for the next phase, the test execution and evaluation. (2) The program is compiled with the compiler

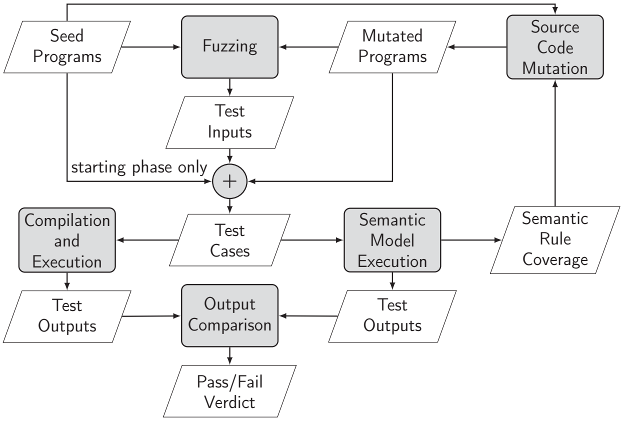

Fig. 1: Overview of the data flow of SpecTest and executed with test inputs generated by the fuzzer. The final state (i.e., variable valuations) is obtained as the program execution result. (3) ***An executable language semantics*** is executed with the same program and the same inputs, through firing a set of structural operational semantic (SOS) rules. The final state is obtained as the semantic execution result. During the semantic execution, we monitor how frequent each SOS rule is fired in order to identify rarely fired rules. (4) The results of the program and semantic execution are compared in order to assess whether the program (built by a compiler) produces an output which is consistent with the language semantics. If the results are inconsistent, the test case is flagged as a failure. The failure may be either due to a bug in the compiler (or the execution environment of the program, e.g., JVM) or in the language semantics. (5) We rank the SOS rules according to the number of times they are fired and identify the ones which are least fired. Each SOS rule is typically associated with one language feature and thus we are able to systematically identify language features which are least tested. With the information, ***a program mutator*** mutates the seed programs so that the corresponding language features are introduced systematically into the programs. In contrast to classical mutation testing [33], which ensures the quality of test suites, we apply mutations to generate more and better test cases. (6) We then repeat from step (1), and the process continues until a user-specified timeout is triggered. The output of SpecTest includes a set of passed/failed test cases as well as a report on the semantic coverage, i.e., the number of times each SOS rule is fired.

It should be noticed that there are three main components in SpecTest, i.e., the executable program semantics which serves as oracle, the program fuzzer, and the program mutator. We present details of these components in the following.

## 2.2 The Oracle

The oracle is an executable semantics of the programming language. That is, the oracle encodes the language semantics in the form of small-step SOS rules. Given a program (and necessary inputs for the program), the oracle is capable of executing the program according to the language semantics to produce the expected output, without going through the compiler to be tested.

Creating an executable semantics for a programming languages is not trivial. It requires experience as well as effort. Nonetheless, it is desirable to have one

```
rule I1 + I2 => I1 +Int I2
```

```
rule if ( true) S else _ => S
```

```
rule [Allocate-Global-NonArrayType]:
<k> #allocate(N, CN, #varInfo(X:Id, E:Value, T:NonArrayType, #storage, L)) =>
      . ...</k>
<account>
   <acctID> N </acctID> <contractName> CN </contractName>
   <acctEnv> CONTEXT:Map => CONTEXT[X <- #storedVar(Slot +Int 1, T, #storage,
         1)] </acctEnv>
   <acctStorage> STORAGE:Map => STORAGE[Slot +Int 1 <- E] </acctStorage>
   <acctSlots> Slot => Slot +Int 1 </acctSlots> ...
</account>
```

Fig. 2: Example SOS rules for Solidity [44]

because it provides a reliable way to check the correctness of compilers, and it will save time and effort in the long term since it effectively reveals ambiguities, inconsistencies and incompleteness. Many researchers have realised the importance of executable language semantics and have built foundations that we can work with, like the K framework [50], Redex [37], or Ott [51]. There are already executable semantics for many programming languages, like C, JAVA, JavaScript, or Solidity, which represent a strong oracle for compiler testing.

It is conceivable and in fact confirmed by our experiments that the oracle itself can be buggy due to human errors in encoding the language semantics or due to ambiguity in the language semantics in the first place. However, even a potentially buggy executable semantics is much better than none for the following reasons. First, during the above-mentioned process, SpecTest is able to identify bugs in the oracle, which helps to improve the language semantics. Second, bugs in the semantics are overall less likely compared to compiler bugs since the compiler must not only implement the semantics but also handle sophisticated code optimisations, which are known to be error-prone.

In this work, we apply the K framework [50] as a basis for our oracle. The K framework provides convenient notations for defining language semantics or type systems based on rewriting rules, configurations, and computations. It comes with a range of supporting tools, like a parser, an interpreter, or a program verifier, which enable the execution of the specifications. In short, it combines the functionality of both the compiler and the runtime environment. Encoding small-step SOS rules in the K framework is relatively straightforward. For example, Fig. 2 shows three (simplified) rules defined for Solidity (i.e., a language for programming smart contracts) programs. In particular, the first rule shows how simple addition should behave for Integers, given the existing k construct for addition `+Int`. The second example is a rule for an if conditional statement, where the condition is true and the result is the then-branch. Not all rules are simple though. The third example is a rule for the storage allocation of a global non-array variable. In general, the rules become more complex for sophisticated language features such as concurrency or higher order functions.

In this work, we adopt and extend the K semantics for Java [24] and Solidity [34,44] to implement SpecTest. The K semantics for Solidity, called KSolidity, has currently 304 rules. The K semantics for Java, called K-Java, has 1385 rules. K-Java was developed for an earlier version of Java (1.4) and some rules are deprecated or unreachable. Our extension to these existing efforts concerned mainly two aspects, i.e., extending them with proper interface and conversion so that they work with other components in SpecTest; and introducing a measurement feature for semantic coverage. For example, we enhanced the coverage engine of the old K version for K-Java, and we added a visualisation of the covered rules.

Given a test case (in the form of a program with inputs), the executable semantics is used as follows. First, the test case is executed using the built-in execution engine of the K framework which fires the SOS rules one by one. The final variable valuations are captured as the result of the test case. For instance, for Solidity, we capture all the persistent states in the blockchain network (which includes addresses, their balances and the values of storage variables). This testing result is turned into an assertion in the test case. The test case with the assertion is then executed using the compiled program. If the assertion fails (e.g., the value of at least one variable is different), a bug is revealed.

Simply applying the above-mentioned steps to test compilers would not be comprehensive. That is, existing seed programs often use a limited set of common language features and thus would not be able to test the compiler extensively. In fact, our experience on testing the Solidity compiler with existing smart contracts suggests that many smart contracts are suspiciously similar. As a result, the test cases would only exercise a limited set of semantic rules and thus would miss those bugs in the part of the Solidity compiler that encodes the remaining semantic rules. While collecting a large set of seed programs would likely be helpful, the larger problem at stake is whether there could be a certain quantitative measurement on the comprehensiveness of the test cases and whether we can use the measurement to guide the generation of new test cases? SpecTest's answer to this question lies in the design of the mutator and the fuzzer.

### 2.3 The Mutator

Due to the high complexity of modern compilers, it is important that a meaningful coverage criterion is applied for compiler testing. Existing approaches either are not concerned with coverage or they use coverage criteria which are not ideal for compiler testing. Hence, we introduce our novel semantic coverage.

**Definition 1.** *Given $R$ is the set of all semantic rules of our specification, $T$ is the set of our given test programs, $I_t$ is the set of all possible inputs for the test program $t \in T$, and $cover(t, i, r)$ is a predicate that is true when there exists a test program $t$ and a test input $i \in I_t$ for $t$ and they are able to fire the semantic rule $r$ of our specification; our semantic coverage can be defined as follows:*

$$\forall r \in R : \exists t \in T : \exists i \in I_t : cover(t, i, r)$$

This means that to achieve semantic coverage (or at least increase it), it is not only important that we have good test programs, but also the test inputs for these programs are essential. In order to produce good test programs, we apply our mutations that inject language features to specifically target the uncovered rules as we will explain in detail in the following. The coverage of all rules $r \in R$ would give us full semantic coverage, but in reality this is often infeasible, hence we also depict it as the percentage of rules that are covered.

In SpecTest, we achieve high semantics coverage with the following two synergistic parts. First, we design and implement a mutator which systematically introduces less-exercised language features into the test programs automatically. Second, we design and apply powerful fuzzing techniques to generate program inputs to exercise all statements including the less-used features in the test programs. The latter can be achieved with fuzzers optimised for existing code coverage criteria such as branch or statement coverage.

We believe that a comprehensive test suite for a compiler must cover all relevant aspects of the language semantics, and semantic coverage offers such a measurement. The above definition simply measures whether a rule is fired or not. It might be meaningful to further measure the context in which each SOS rule is fired (as certain bugs might only be triggered when a rule is fired in a certain context), which we leave as future work.

To achieve high semantic coverage, SpecTest employs a two-part solution. Given the oracle's feedback on which SOS-rules are not fired (or least fired), the language features which are associated with the SOS rules are identified. This is straightforward as each SOS rule is associated with a specific language construct. For instance, when the first rule of Fig. 2 is not fired, then this would highlight that our test programs contain no addition between Integer variables. Next, the mutator takes the information and systematically mutates the seed programs to introduce these less-tested language constructs.

The mutator is a code mutation engine which is designed to automatically mutate a given source program to generate new programs (i.e., test cases for the compiler). Existing mutation approaches [38,41,55] for compiler testing already applied mutators to generate test programs, but they mutate based on simple algebraic rules and are not systematic. For instance, equivalence modulo inputs (EMI) [41] works by injecting code into seed programs with the aim to achieve a high difference in the control- and data-flow compared to the original seed program in order to produce diverse test programs. In comparison, our mutator is designed to maximise semantic coverage.

Implementing the mutator is not trivial. For SpecTest, the mutators for Solidity and Java were implemented based on existing parsers through code instrumentation. That is, given a language feature and a source program, the mutator first parses the source program to build an AST. Afterwards, it identifies potential locations in the AST for introducing the features. Lastly, it systematically applies a mutation strategy specifically designed for the language constructs to inject them at all possible or specific pre-defined locations. In the following, we introduce three mutation strategies as examples.

We investigated features that were specific for Solidity. For example, one mutation introduces modifiers for functions, which define conditions that must hold when a function is executed. Listing 1.1 shows a smart contract with modifiers written in the Solidity language. Unlike traditional programs, smart contracts cannot be modified once they are deployed on the blockchain. As a result, their correctness is crucial. So is the correctness of the compiler since the compiled programs are deployed on the blockchain. Furthermore, the Solidity compiler

```
1 contract AccessRestriction {
2  address public owner = msg.sender;
3  //default modifier:
4  modifier onlyBy(address account){
5   require(msg.sender==account, "Sender not authorized");
6   _;  } //injected modifier:
7  modifier cgskst(address value){
8   require(value == address(0x0),"");
9   _; } //injected modifier:
10 modifier cbhsmo(address value){
11  require(value == address(0x0),"");
12  _;  } //injected modifier:
13 modifier nlwxmv(address value){
14  require(value == address(0x0),"");
15  _;
16 }//Make newOwner the contract owner:
17 function changeOwner(address newOwner) public onlyBy(owner) cgskst(address(0x0)) cbhsmo(address(0x0)) nlwxmv(address(0x0)){
18     owner = newOwner;
19 }}
```

Listing 1.1: Simple modifier example

```
bibt4QkDIfJ: {
  bsJxhbtSJBu: {
      bHhq230wDjZ: { try {
          bEdqZ33tKi9: {
            bVm9tCxbul4: {
              if (i >= 5){ break; }
                break bEdqZ33tKi9;
            }  }
        }catch(RuntimeException e){
          bQ2yucCPLQr: {
            System.out.print("X");
            break bQ2yucCPLQr;
} } } } }
```

Listing 1.2: Labelled block mutation

```
contract Test {
  function testFunc(int a)
    public pure returns (int) {
    int result = a + a++;
    //produces 3 when a is 1
    return result;
} }
```

Listing 1.3: Simple contract example has been under rapid development and there are unique language features with sometimes confusing semantics. Thus, it is a good target for evaluating the effectiveness of SpecTest. In this example, the modifier `onlyBy` ensures that the function `changeOwner` can only be called when the address of the contract owner is used. By integrating various dummy modifiers (Lines 7, 10 & 13) into our seed contracts and by adding them to functions (Line 17), we noticed that an older version of the Solidity compiler crashed in some cases, when more than a certain number of modifiers are used. Such a case is difficult to find with normal tests, since it is rare to use multiple modifiers for a function. Given that a less-fired SOS rule is concerned with the modifier construct in Solidity, to introduce modifiers, the mutator scans through the AST for function declarations. For each function declaration, the mutator randomly adds one or more modifiers.

We also introduced specific mutations for Java. For example, our experiments showed that semantic rules associated with labels were not fired. Hence, we introduced mutations that target these rules, e.g., a mutation that injects labelled blocks, which is a special and rarely used feature that allows an immediate exit of a block with a break statement. This mutation is illustrated in Listing 1.2, where we injected labelled blocks and breaks (with these labels) into a seed program.

Both for Solidity and Java, we noticed that there are various rules in the K specifications (i.e., 11 rules Java and 17 for Solidity) concerning mathematical expressions that were not covered, e.g., computations with hex-values. In order to cover these rules and to cover unusual usages in different contexts, we relied on a random approach in contrast to the other mutations where we injected code at specific places. We developed mutations that produce a variety of mathematical expressions combining various language features, like operations containing variables with different data types, hexadecimal, octal or binary literals, pre- and postfix increment/decrement (`++`/`--`), bitwise and bitshift operators, various combinations of unary operators and arrays. A simplified example of a mutation produced with this strategy is shown in Listing 1.3. It can be seen that the increment operators (++) is used in an unusual context within a mathematical expression. Our experiments showed that the computation produced unexpected results, i.e., we found an issue with the computation order that caused the increment to be executed first, although it should be executed last [19].

### 2.4 The Fuzzer

By injecting specific language features into the seed programs, the mutator increases the likelihood of firing uncovered or poorly covered SOS rules during the test execution. The fuzzer is a fuzzing engine which generates test inputs for a given program. The generation is based on optimization (e.g., using genetic algorithms). One of the required inputs for the fuzzer is a set of seed source programs. Such source programs are often abundant. For instance, there are thousands of Solidity programs (contracts) on EtherScan.io. The fuzzer takes these contracts as input and generates test inputs for each contract. During this process, the fuzzer sets up a test blockchain network, deploys the contracts, and generates a sequence of transactions which invoke functions.

For Solidity, we applied an existing smart contract fuzzer called sFuzz [46] that works with a new adaptive fuzzing technique for maximising the branch coverage. sFuzz uses an optimised version of a technique called American Fuzzy Lop (AFL) [59], for producing inputs that can achieve a high branch coverage. It includes various test oracles for the detection of general vulnerabilities, like Integer overflows, or smart contract specific vulnerabilities, like a gasless send [48]. We applied sFuzz to maximise the coverage of our test programs to cover our injected features. For our injected features, the coverage was usually easily achieved. However, for other cases or to minimise the test inputs, it might be necessary to customise the fuzzer to specifically target newly added language features. For example, during the mutation, we can record which parts of the contracts have been mutated and prioritise those parts during fuzzing. For Java, we did not apply a fuzzer, because the majority of our seed programs were simple in nature. A single run produced full coverage in almost all cases.

## 3 Evaluation

We have implemented SpecTest for two compilers, a compiler for a general purpose language (Java) and one for a new domain-specific language (Solidity). In the following, we design multiple experiments to systematically answer the following research questions (RQ).

- *RQ1: How effective is our proposed method in finding bugs or inconsistencies?* This is important since the primary aim of SpecTest is to provide a systematic way of generating a test suite for identifying compiler bugs.
- *RQ2: What kind of bugs and inconsistencies can be found?* To further motivate the usage of SpecTest, it makes sense to point out what issues can be found. In particular, we would like to check whether indeed there are compiler bugs associated with less-fired SOS rules.

– *RQ3: To what extent can the coverage of rules within the language specification be increased with specific mutations?* The semantic rule coverage is one of the core aspects of SpecTest for finding bugs. Therefore, it is important to investigate to which extent we can increase this coverage.
– *RQ4: How much effort is it to apply SpecTest?* When a tester is considering a testing method, the effort usually plays a big role. To create a good basis for a decision, we discuss the effort of applying SpecTest to two compilers.

### 3.1 Test Setting

As seed programs, we used existing tests cases of K-Java [24] and KSolidity [34,44]. KSolidity is still under development, which means that we could not test all features or a large set of contracts, but it was already sufficiently developed to support many interesting cases. K-Java supports most features of Java 8, but it also has limitations, i.e., it was implemented in an old version of the K framework, which did not focus on performance. Hence, we used seed programs without imports of libraries. We do not regard this as a limitation since small programs have advantages, e.g., they are easier to debug and it reduces the time for test case minimisation. Moreover, it is well-known that many bugs can be revealed by small test cases [32], which are also common in traditional testing.

For Solidity, we had 37 seed programs that were part of the KSolidity project due to its early stage. Hence, it makes sense to apply SpecTest since it enables the generation of more test programs in a systematic way. Our mutator for Solidity is written with about 5,300 lines of Java code. In each test run, we applied one of our mutations (or in some cases also combinations) to the seed programs. We applied sFuzz to the mutated contracts and then converted the resulting test cases in a usable form for KSolidity. We primarily tested the Solidity compiler version 0.5.13, but initially also older versions. In some cases, we had to apply Truffle tests [21] (v5.1.10) and for debugging we used Remix [18], which facilitates a step-by-step exploration of the contract bytecode.

For Java, we applied 756 seed programs and our mutator has about 6,100 lines of code. The mutations were similar as explained before. In contrast to Solidity, we did not need a sophisticated fuzzer since the mutated Java programs were covered easily. Our focus was Java 13 (openjdk 13, 2019-09-17, RE build 13+33-Ubuntu-1), but we also tested older versions (11 and 8). For the mutator, we applied JavaParser 3.14.3 for parsing the programs and for injecting mutations.

The experiments for Solidity were performed on a Dell X1 Carbon with an Intel i7-8565U CPU with four 1.80GHz cores and 16 GB RAM, for Java on a PC with an Intel i7-7700 CPU with four 3.60GHz cores and 64 GB RAM.

### 3.2 Experiment Result

We ran more than 30,000 test cases for Java, which had a total execution time of about three weeks. For Solidity, we ran more than 50,000 test cases with a total execution time of about two weeks. Details about the distribution of the run time will follow below. The execution times are not exact numbers, since the experiments sometimes were stuck due to out of memory exceptions, not enough space, etc. Unfortunately, we could not fully resolve such issues, because many mutations inject features with random aspects into the diverse seed programs. This caused various unpredictable situations, like endless loops or too large data structures. By adopting our mutator, we greatly reduced the number of such situations, but we could not remove all rare cases.

*RQ1: How effective is our proposed method in finding bugs or inconsistencies?* We discovered issues and bugs both for Solidity and Java. Some of these issues were not found within the compiler or the runtime environment, but within the language semantics. Fixing such issues is also essential, since improving the specification is an important aspect of testing.

In total, we found six issues for the Solidity compiler [19,10], two were related to error/warning messages [7,13], and three of the other issues might have the same cause, i.e., the execution order. For KSolidity, we found eight issues, six of them were related to unimplemented features. For Java, we found four issues with the compiler [2,5], two of which were concerned with error messages [6,12], and we discovered 13 issues with K-Java [14,15,11,9,8,3,1,16] (eight issues or bugs, one warning related issue, and four minor issues, like a wrong output representation [16]). More details about the different types of issue follow below.

Our experiments showed that SpecTest is able to reveal issues, inconsistencies and bugs. These issues were not only found in the compiler, but also in language semantics (which are developed independently by other groups with dedicated effort). One might argue that finding bugs/issues in the language semantics is not as meaningful as finding bugs in the compiler. We believe that it is also crucial to ensure the robustness of the semantics since in general the quality of the tests or specification are essential for the overall robustness of software. SpecTest was able to find various inconsistencies and bugs in the specifications, which is important for the specification developers, as well as issues in the compilers. We have spent effort on confirming our findings and out of the 31 issues, we submitted 19 to the corresponding git repositories and reported the other issues to the developers or to a bug reporting system. For 13 issues, we received a confirmation or the developers mentioned that they will investigate and fix them.

An aspect that might have limited the effectiveness, is that we did not fully apply our method for Java, since we only tested simple seed programs and did not use fuzzing. We believe that the issues we found still showed that our method was reasonably effective, even though we only partially applied it. Using the full extend of SpecTest for Java might require a more powerful specification, which is a potential topic for future work. Moreover, it should be mentioned that KSolidity is still being developed and not as stable as the Solidity compiler (or runtime environment), since much more effort was invested into its development. This is similar for K-Java, and Java in general is robust due to its maturity.

*RQ2: What kind of bugs and inconsistencies can be found?* We categorise our findings into three categories as illustrated in Table 1, i.e., (1) normal issues, bugs and missing features, (2) issues related to warning or error messages, and (3) minor inconsistencies or issues, like a small discrepancy in the output, e.g.,

`-0e+00.0` instead of `-0.0` [16]. Additionally, we differentiate whether the origin of an issue was the compiler or the specification, as illustrated by the rows of Table 1.

Table 1: Found semantics and compiler issues

| | Solidity | KSolidity | Java | K-Java |
|---|---|---|---|---|
| **Normal issue or bug** | 4 | 8 | 2 | 8 |
| **Warning or error message related issues** | 2 | - | 2 | 1 |
| **Minor issues** | - | - | - | 4 |
| **Total** | 6 | 8 | 4 | 13 |

The most interesting issues that we found were the ones concerning the wrong computation order in Solidity. The cause of these issues were actual semantic errors within the compiler. Moreover, we also found various issues with error or warning messages. Such issues might seem trivial, but it is important to fix them since meaningless error messages can cause a huge waste of debugging effort. The bugs we found in the specifications had multiple sources, like the syntax parser, wrong semantic rules, partially implemented rules, or rules applied in a wrong context. Although K-Java and KSolidity had already many manual tests, we showed that SpecTest was able to discover many inconsistencies and bugs. In the following, we present example issues from the mentioned categories.

*Solidity Findings.* One of the issues [19] that SpecTest identified was that there were wrong results, when we tested expressions with different assignment operators. The behaviour can be observed in the following example, where the increment operator is applied at first, but should be applied in the end.

```
int a = 2;       a *= 1 + a++; //results in 9 but should be 6
```

A potential cause might be a wrong computation order. This issue was found since some SOS rules for assignment operators were uncovered. By creating mutations that target these rules, we could generate expressions like in the example which led to the discovery of the issue since the oracle predicted a different result.

An inconsistency regarding an error message [13] was revealed when we tested computations with different data types. As illustrated below, we discovered that it is possible to add int variables with different bit sizes, but an error is produced if an int_const is added to an int variable with a smaller bit size.

```
int8 a = 10; int16 b = 234;
int c = b + a; // works
int c = 234 + a;//TypeError: Oper. + incompatible with types int_const & int8
```

In this case, our oracle performed the computation without an error, but the Solidity compiler produced a type error. For KSolidity, we found an incorrect overflow behaviour for computations, and that there is no support for numerous language features, like increment operators.

Additionally, we applied our Solidity truffle tests to the Conflux blockchain [17], which is a new alternative for Ethereum. It basically can be seen as another runtime environment for Solidity contracts. With our tests, we were able to reveal a bug in the testing environment that resulted in incorrect results when we injected formulas with unary and bitwise operators [4].

*Java findings.* Our experiments showed that there is an inconsistency [1,2] when casts from double and long variables to Integers are performed. These casts are handled differently by Java when an overflow occurs, i.e., in the following code the results will be the maximum Integer for the double cast and bits will be cut off for the long cast. In K-Java both casts produce the same result, i.e., bits will be cut off. Although this behaviour is documented in the language specification and already others were wondering about this issue, we believe that the approach of K-Java is more consistent, and we are still waiting for a comment of the Java team about the motivation to handle these cases differently.

```
System.out.println(((int)2147483648L));  // -2147483648
System.out.println(((int)2147483648.0)); //  2147483647
```

A problem we found for the Java compiler [6] is a missing error message when a computation with a long and a double variable is performed. Normally, an incompatible types error is produced as illustrated in the following code, but the error does not occur when the same computation is done with an += operator.

```
long a = 1L + 0.1 * 3L; // produces error: incompatible types: possible lossy
long b = 1L;            //                 conversion from double to long
b += 0.1 * 3L;          // no error is produced
```

We discovered that K-Java has an issue with the modulo operator [14]. The computation is wrong for all negative doubles and floats, i.e., it produces inconsistent values compared to Java and compared to the same computation with Integer values. This is illustrated in the following examples.

```
System.out.println("-8   %  3   = "+(-8   %  3));  //K-Java and Java return -2
System.out.println("-8.0 %  3.0= "+(-8.0 %  3));  //K-Java 1.0 Java -2.0
System.out.println(" 8   % -3   = "+( 8   % -3));  //K-Java and Java return 2
System.out.println(" 8.0 % -3.0= "+( 8.0 % -3.0));//K-Java -4.0 Java 2.0
```

In general, we found most issues, when we injected mathematical expressions into the seed programs. This was an interesting finding for us, since these expressions are a major component of all programming languages, and we assumed it would be straightforward to develop a specification for them. However, it turned out that many interesting and ambiguous situations can occur when various combinations of operators, variables and literals are tested.

*RQ3: Can SpecTest effectively improve semantic coverage?* The objective of SpecTest is to systematically generate a test suite for achieving better semantic coverage. In order to evaluate the coverage, we conducted the following experiments. We identified the semantic rules that were least covered by the existing tests for Solidity and Java, and then applied SpecTest systematically (with specific mutators) and measured the improvement in terms of semantic coverage.

First, we evaluated the semantic coverage criterion that is achievable with the original seed programs of K-Java and KSolidity to have a reference value for the comparison with the mutated test programs. Table 2 shows a comparison of the coverage from the original test cases from K-Java to our mutated test cases. The rule coverage of this early version of the K framework of K-Java is rudimentary. Hence, we could only measure the covered lines and characters of the rule files, and many of these files were already fully covered due to redundant or unreachable rules. Nevertheless, we were able to identify various uncovered rules in four of the files, and we produced mutations that covered these rules.

KSolidity was built with a new version of the K framework, which has a better measurement of the rule coverage. Since the development of KSolidity is still ongoing, we focused on the completed features, like conditions, loops, arrays, structs, simple transactions, or mathematical expressions, and managed to increase the coverage. Even with just these features, we found meaningful bugs. The coverage improvements compared to the original seed programs are illustrated in Table 3. There were partially implemented features which could not be fully covered. The coverage of the completed features was considerably improved.

Table 2: Comparison of the covered rules between the K-Java tests (Default) and our mutated test cases

| File | Default | | Mutants | | Difference | |
|---|---|---|---|---|---|---|
| | Char | Line | Char | Line | Char | Line |
| folding.k | 93.04 | 93.89 | 93.04 | 93.89 | - | - |
| unfolding.k | 91.84 | 94.55 | 91.84 | 94.55 | - | - |
| process-class-decs.k | 89.07 | 92.95 | 89.07 | 92.95 | - | - |
| expressions.k | 72.30 | 78.74 | 86.58 | 89.92 | 14.28 | 11.18 |
| process-comp-units.k | 83.39 | 86.03 | 83.39 | 86.03 | - | - |
| static-init.k | 81.20 | 82.35 | 81.20 | 82.35 | - | - |
| process-class-members.k | 80.65 | 83.53 | 80.65 | 83.53 | - | - |
| statements.k | 80.51 | 82.38 | 80.51 | 82.38 | - | - |
| new-instance.k | 79.59 | 82.41 | 79.59 | 82.41 | - | - |
| method-invoke.k | 79.44 | 80.74 | 79.44 | 80.74 | - | - |
| api-core.k | 61.77 | 63.37 | 78.74 | 81.82 | 16.97 | 18.45 |
| var-lookup.k | 77.52 | 79.41 | 77.52 | 79.41 | - | - |
| process-type-names.k | 76.56 | 75.76 | 76.56 | 75.76 | - | - |
| expressions-classes.k | 73.03 | 65.00 | 73.03 | 65.00 | - | - |
| process-local-classes.k | 67.62 | 72.12 | 67.62 | 72.12 | - | - |
| process-anonymous-classes.k | 66.79 | 81.52 | 66.79 | 81.52 | - | - |
| arrays.k | 62.07 | 66.90 | 62.07 | 66.90 | - | - |
| api-threads.k | 35.51 | 39.04 | 41.43 | 47.01 | 5.92 | 7.97 |
| syntax-conversions.k | 40.65 | 42.42 | 40.65 | 42.42 | - | - |
| literals.k | 29.19 | 34.31 | 38.73 | 42.72 | 9.54 | 8.40 |

We have shown that our mutations can increase the rule coverage both for K-Java and KSolidity. Our close investigation shows that the increase in coverage requires non-trivial programs (e.g., programs that specifically include missing language features) which are unlikely to be generated without our mutator. It is worth mentioning that writing mutations for the uncovered rules lead to the discovery of many issues. Moreover, the mutations that targeted specific semantic rules or language features could generally increase the coverage instantaneously with a single test, but we still applied them to all seed programs, and we also used general mutation operators to produce mutants for many different situations.

*RQ4: How much effort is it to apply SpecTest?* To answer this question, we analysed the effort required to apply and implement SpecTest for Java and Solidity. It consists of two parts, the effort of applying SpecTest once it is developed, and the implementation effort. The latter one consists of three parts, the effort for developing the oracle, the mutator and the fuzzer. The goal of this analysis is to understand how generalisable SpecTest is to a new programming language.

Applying SpecTest after the implementation has the following timing requirements. Both for Solidity and Java, the mutant generation took only a few seconds. For Solidity, we set a timeout of 2 min per contract for fuzzing and it took on average 24 min to finish all 37 contracts. Usually, 40–45 test cases were created by the fuzzer (normally multiple per contract depending on the mutation). Most test cases were executed by KSolidity within a minute, but there were outliers which did not terminate even after hours. Hence, we used a timeout of 5 min. On average, the testing time of KSolidity was 37 min (when five runs with different mutations were considered). For Java, we did not apply a fuzzer due to the simplicity of the seed programs. We executed the 756 test programs directly with K-Java, which took on average 3 hours and 51 min for an introduced mutation (for five runs with different mutation types).

Table 3: Comparison of the covered rules between the original KSolidity tests (Default) and our mutated test cases

| File | Default | Mutants | Difference |
|---|---|---|---|
| function.k | 86.21 | 86.21 | - |
| expression.k | 92.31 | 95.38 | 3.08 |
| solidity-syntax.k | 62.50 | 72.12 | 9.62 |
| statement.k | 92.86 | 92.86 | - |
| contract.k | 94.12 | 94.12 | - |
| driver.k | 49.15 | 49.15 | - |
| solidity.k | 50.00 | 50.00 | - |

We now discuss our development efforts and the time requirements of the implementation of SpecTest for a new language. In our case, the most effort went into the development of the mutator and the supporting tooling, like translators. The implementations for both Solidity and Java took about two to three months each. It should be noted that this time depends on the availability of existing tools, like a language parser or fuzzer. For this work, we relied on pre-existing language specifications, which helped to reduce the overall effort, but as mentioned they came with limitations, which caused additional efforts. Writing a specification for a new programming language is not trivial. Based on past experiences, we assume that it takes about six to 12 months depending on the complexity of the language. Given the many recent efforts on developing executable language semantics, we believe that SpecTest provides a good way to better utilise these existing specifications for systematic compiler testing.

To summarise, the implementation effort of SpecTest is about two to three work months mainly for the mutator, if there is an existing specification and a fuzzer. The application of our method in terms of run time is about a few hours for a single mutation. Further increasing the number of seed programs, and performing a reasonable number of mutations increases this time to a couple of days or weeks, when the tests are only executed on one machine. Even though this seems like a lot of effort, we believe our method is still worthwhile, since it will pay off eventually, especially considering all the effort that can be required for releasing a new compiler version, when serious bugs are discovered. Moreover, our method can be easily accelerated by distributing it to multiple machines.

As mentioned before, the implementation effort for our method was about two to three work months. This is about the time that is needed for the mutator and for other minor tools. It does not include the effort for creation of the language specification or the fuzzer. There are already many existing fuzzers that could be adopted for new programming languages, and also numerous language specifications. We especially want to recommend our method for all languages with pre-existing specifications (or when similar specifications exist) since then there is only a small implementation effort, which will soon be mitigated by the advantages of SpecTest. Even when there are no pre-existing specifications for a language, we highly recommend to create one and to adopt our method, since it will save time in the long term.

An effort that should not be underestimated is the time for analysing bugs. It can be troublesome and to find the cause of a bug, due to the complexity of the test cases, i.e., it sometimes took us hours or even days. In such cases, it can be helpful to minimise failing test cases. There are numerous techniques, like delta debugging [62] or program slicing [58], which can reduce the debugging effort, and integrating them into SpecTest would be interesting for future work.

### 3.3 Threats to Validity

A threat to the validity of our evaluation might be that we did not show a comparison to other compiler testing methods. A comparison might be interesting, but our main goal was to show the general applicability and usefulness of SpecTest for different compilers. It would not be fair to compare SpecTest to other testing techniques that focus on different types of bugs, e.g., it might be much easier to find simple parsing errors caused by unusual characters (with techniques, like fuzzing).

One might argue that the test size we used is too limited, which might be a potential threat to the validity of our evaluation. It is true that it would make sense to apply more seed programs and to continue mutating and testing for an extended period of time. However, due to restrictions of KSolidity and K-Java, a larger set of seed programs was not supported, and due to a limited time and computing budget, we did not execute more tests. Nevertheless, we believe that our test size was reasonable, since it allowed us to reveal various issues and bugs.

Another threat to the validity of our evaluation might be that we should not have just relied on existing specifications, where we cannot be sure about their quality. It is true that we might have more confidence in a specification that we created, but since SpecTest checks the correctness of compilers as well as specifications, we have trust that our specifications had a reasonable quality.

## 4 Related Work

Compiler testing is a broad research field with a range of techniques that target, e.g., the test case generation [49,31,23] or the oracle problem [22]. Several surveys give an overview of these methods [56,26,39,25]. Our study however shows that existing approaches suffer from two weaknesses. They do not apply a test case generation that can extensively cover rare language features, and they often rely on weak or limited test oracles. The test case generation often works with standard code coverage criteria concerning compiler components. For example, Zelenov and Zelenova [61] applied a BNF grammar as a model and produced test cases according to, e.g., code or functional coverage of a syntax analyser. A method based on the coverage of context-free grammar rules was presented by Purdom [49], but it only targets the parser of the compiler. Kalinov et al. [35,36] defined coverage criteria based on a statement machine specification. In contrast to our work, they do not identify rare language features by analysing semantic rule coverage, and they do not construct their test programs via code mutation.

Various compiler testing methods work without any coverage by just randomly generating test cases according to a grammar, which defines valid programs [52,60]. There are also techniques that use mutation for producing test cases [38,41,55]. For example, Le, Sun, and Su [41] produced mutants that should have the same behaviour as the original programs in order to find cases where the behaviour diverges. However, in contrast to our work, they are not considering a semantic coverage for less used language features.

Several attempts have been presented to answer the oracle problem for compiler testing. In the simple case of positive/negative testing, an oracle only tells whether a program is compilable. When a test program is compiled, the result is checked to see if it matches the expectation of the oracle. A match means a successful compilation. Otherwise, there may be a bug. For example, Zelenov and Zelenova [61] illustrated a specification-based approach for generating positive and negative tests. Such approaches are limited to testing the syntax parser.

In the line of work on differential testing compilers [45], the oracle is defined as consistency among two or more compilers for the same language. In this method, the same test programs are given to multiple compilers and the results are compared. If there is a difference then a bug in one of the compilers or an ambiguity in the language is found. There exist different versions of differential testing as explained by McKeeman [45]. Cross-compiler testing [52] is a technique that works by contrasting a new compiler against a pre-existing compiler that has the same specification. When the same test programs are executed with both compilers, a different result can reveal a fault in the new or pre-existing compiler. Sometimes this technique is also called randomised differential testing [60], because the test programs are usually generated randomly, e.g., based on a grammar. Another differential testing technique is cross-optimisation testing, where programs compiled with different optimisations implemented for the same compiler are contrasted to find bugs. Le, Sun, and Su [42] presented such a technique for stress testing link optimisers. Their method generates random test programs and injects various function calls into different code regions in order to increase dependencies between procedures, and it also randomly selects different optimisation levels to produce challenging tests for the optimiser. Cross-version or regression testing is another differential testing method that tries to find bugs by comparing different versions of the same compiler. For example, Sun, Le, and Su [54] developed Epiphron, a tool that generates random test programs to find inconsistencies with the debug information, like missing warning messages, in different versions of the same compiler. Such approaches work only if there are multiple relatively mature compilers for the same language. In contrast to these techniques, SpecTest works with a formal language specification which is especially useful when no compilers could be used as a reference. Moreover, different compilers or compiler versions for the same language might still suffer from the same bugs, which is unlikely for an independent specification.

There are approaches that assume the existence of a reference compiler, i.e., the oracle is an existing formally proven compiler. For example, Leroy [43] presented CompCert, a compiler for a subset of C, which was verified with the proof assistant Coq. However, there are usually no such compilers for a newly developed language and the existing ones cover only subsets of languages since formally proving a compiler is extremely challenging.

For metamorphic testing [57], the oracle is defined as certain algebraic properties of the compiler. For instance, one such property explored in the compiler testing technique called equivalence modulo inputs (EMI) [40,55] is that a modification on a program part which is never executed should not alter the result.

Based on this simple oracle, EMI works by randomly pruning dead code (i.e., code which is not executed given a certain program input) or by randomly inserting or removing instructions from dead code based on a Markov Chain Monte Carlo method. Such approaches are limited to identifying bugs which violate the algebraic properties. Hence, they are not able to find deep semantic errors.

The closest related work to SpecTest was proposed by Kalinov et al. [35,36], where a language specification in the form of abstract state machines and montages is used as an oracle. With this specification, they compare the expected output from the specification to that of a compiled program in order to check whether there are compiler bugs. This approach is limited by the choice of the specification language and it quickly becomes infeasible, because the computation time is too high. Moreover, it is not concerned with semantic coverage.

To demonstrate the limitations of the closely related methods, we come back to the example of Sect. 2, i.e., we discussed a bug with the increment operator that we discovered during our analysis of the Solidity compiler.

```
int a = 1;       int result = a + a++; //produces 3, but it should be 2
```

In this example, the compiler had an issue with the computation order, which resulted in wrong results. Existing approaches, like EMI or differential testing might be able to detect such issues, but with EMI it is difficult to find mutations that lead to such cases. The same is true for differential testing and there is also a high chance that different compiler versions have the same faulty behaviour for such a case (e.g., all versions of the Solidity compiler had this issue).

## 5 Conclusion

We have demonstrated our novel compiler testing technique called SpecTest that targets less-used language features. SpecTest is based on three components: an executable language specification, a fuzzer for generating test inputs, and a mutator which generates new programs by injecting rare language features. Comparing the abstract execution of the specification to the concrete execution of a compiled program enables our method to find deep semantic errors as well as inconsistencies and issues in the specification.

We evaluated SpecTest by applying it to two programming languages: Java and Solidity. The results are encouraging. We discovered various issues concerning the compilers and the language specifications. Some of them helped to improve the quality of the compilers and many will enhance the specifications.

In the future, we plan to further explore the generality of SpecTest for other languages, and we intend to consider different types of executable specifications.

**Acknowledgments**

This research is supported by the National Research Foundation Singapore under its NSoE Programme (Award Number.: NSOE-TSS2019-03).
